# Intraoperative Faszientraktion (IFT) zur Behandlung großer ventraler Hernien: Eine retrospektive Analyse von 50 Fällen

**DOI:** 10.1007/s00104-021-01552-0

**Published:** 2021-12-14

**Authors:** Henning Niebuhr, Zaid Omar Malaibari, Ferdinand Köckerling, Wolfgang Reinpold, Halil Dag, Dietmar Eucker, Thomas Aufenberg, Panagiotis Fikatas, René H. Fortelny, Jan Kukleta, Hansjörg Meier, Christian Flamm, Guido Baschleben, Marius Helmedag

**Affiliations:** 1 Hamburger Hernien Centrum, Eppendorfer Baum 8, 20249 Hamburg, Deutschland; 2 Faculty of Medicine, Department of Surgery, University of Tabuk, Tabuk, Saudi-Arabien; 3 Zentrum für Hernienchirurgie, Vivantes Humboldt Klinikum, Berlin, Deutschland; 4 Chirurgische Klinik Kantonsspital Baselland Bruderholz, Bruderholz, Schweiz; 5 Klinik für Chirurgie, St. Elisabeth-Krankenhaus Köln, Köln, Deutschland; 6 Klinik für Chirurgie, Charité Campus Virchow-Klinik, Berlin, Deutschland; 7 Ordinationszentrum der Confraternität, Wien, Österreich; 8 Klinik für Chirurgie, Hirslanden Klinik, Zürich, Schweiz; 9 Klinik für Allgemein- und Viszeralchirurgie, Sana Krankenhaus, Benrath, Deutschland; 10 Klinik für Allgemein‑, Viszeral‑, Endokrine und Unfallchirurgie, RoMed Clinic, Bad Aibling, Deutschland; 11 Klinik für Allgemein- und Viszeral Chirurgie, St. Elisabeth Hospital, Leipzig, Deutschland; 12 Klinik für Allgemein‑, Viszeral- und Transplantationschirurgie, Universitätsklinik Aachen, Aachen, Deutschland

**Keywords:** Bauchwandhernien, Narbenhernien, Komponentenseparation, Faszienabstand, W3-Hernien, Abdominal wall hernia, Incisional hernia, Component separation, Fascial distance, W3 hernias

## Abstract

**Ziel:**

Es sollen die Effektivität, klinische Praktikabilität und Komplikationsrate der intraoperativen Faszientraktion (IFT) in der Behandlung großer ventraler Hernien untersucht werden.

**Methode:**

In der Untersuchung wurden 50 Patienten aus 11 spezialisierten Zentren mit einem direkt gemessenen Faszienabstand von mehr als 8 cm ausgewertet, die mittels einer IFT (Traktionszeit 30–35 min) unter Verwendung des fasciotens® Hernia-Zugverfahrens (fasciotens Gmbh, Essen, Deutschland) behandelt wurden.

**Ergebnisse:**

Die präoperativ gemessenen Faszienabstände betrugen 8–44 cm, wobei die meisten Patienten (94 %) einen Faszienabstand über 10 cm aufwiesen (W3 nach Klassifikation der European Hernia Society). Der durchschnittliche Faszienabstand wurde von 16,1 ± 0,8 auf 5,8 ± 0,7 cm reduziert (Streckengewinn 10,2 ± 0,7 cm, *p* < 0,0001, Wilcoxon-matched-pairs-signed-ranks-Test). Bei drei Viertel der Patienten konnte eine Reduktion des Faszienabstands um mindestens 50 % erreicht werden, und bei der Hälfte der behandelten Patienten betrug die Reduktion des Faszienabstands sogar über 70 %. Die durch die IFT nach einer mittleren Operationsdauer von 207,3 ± 11,0 min erreichte Verschlussrate betrug 90 % (45/50). Der Hernienverschluss erfolgte in allen Fällen mittels einer Netzaugmentation in Sublay-Position. Postoperative Komplikationen traten bei 6 Patienten auf (12 %). Bei 3 Patienten (6 %) war jeweils eine Reoperation erforderlich.

**Schlussfolgerung:**

Mit der beschriebenen IFT steht ein neues Verfahren für die Bauchwanddehnung bei großen ventralen Hernien zur Verfügung. Die vorliegende Untersuchung zeigt eine hohe Effektivität der IFT bei guter klinischer Praktikabilität und niedriger Komplikationsrate.

## Abstract (en)

**Objective:**

The aim was to evaluate the effectiveness, clinical practicability, and complication rate of the intraoperative fascial traction (IFT) procedure for the treatment of large ventral hernias.

**Method:**

This study evaluated 50 patients from 11 specialized centers with an intraoperatively measured fascial distance of more than 8 cm, who were treated by IFT (traction time 30–35 min) using the fasciotens® hernia traction procedure.

**Results:**

Fascial gaps measured preoperatively ranged from 8 cm to 44 cm, with most patients (94%) having a fascial gap above 10 cm (W3 according to the European Hernia Society classification). The mean fascial distance was reduced from 16.1 ± 0.8 cm to 5.8 ± 0.7 cm (stretch gain 10.2 ± 0.7 cm, *p* < 0.0001, Wilcoxon matched-pairs signed-ranks test). A reduction in fascial distance of at least 50% was achieved in three quarters of the patients and in half of the treated patients the reduction in fascial distance amounted to even more than 70%. The closure rate achieved by IFT after a mean surgical duration of 207.3 ± 11.0 min was 90% (45/50). Hernia closure was performed in all cases with a mesh augmentation in a sublay position. Postoperative complications occurred in 6 patients (12%). A reoperation was required in 3 patients (6%).

**Conclusion:**

The described IFT method is a new procedure for abdominal wall closure in large ventral hernias. The presented results demonstrate a high effectiveness, a good clinical practicability and a low complication rate of IFT.

Bauchwandhernien gehören zu den häufigsten Erkrankungen, die einer (viszeral)chirurgischen Versorgung bedürfen. Die Inzidenz liegt 1 Jahr nach Laparotomie bei ca. 8–16 % [1, 29].

Große Narbenhernien stellen bei einer wachsenden Zahl von durchgeführten Operationen bei immer älteren und zunehmend adipösen Patienten ein wachsendes Problem für den operierenden Bauchwandchirurgen dar. Bruchgrößen von 10–25 cm Querausdehnung und bis zu 30 cm Längsausdehnung sind keine Seltenheit [21–23]. Folgezustände sind Einschränkungen der körperlichen Belastbarkeit, der Darm- und Organfunktionen, Schmerzen sowie kosmetische Beeinträchtigungen [21]. Eine effektive operative Versorgung ist daher für eine Verbesserung der Lebensqualität der Patienten erforderlich [4]. Betrachtet man die Folgekosten, zeigt sich auch eine erhebliche sozioökonomische Bedeutung.

Zur Wiederherstellung der Integrität der Bauchwand kommen – in Abhängigkeit von der Ätiologie, dem Ausmaß des Bauchwanddefektes und dem individuellen Patientenprofil – unterschiedliche Operationsverfahren zur Anwendung [14, 15].

Bei der offenen Komponentenseparation nach Ramirez [2, 25, 27] besteht ein hohes Risiko für Wundheilungsstörungen, Hämatome und Serome [16, 17]. Bei der endoskopisch assistierten Komponentenseparation [3, 17, 24, 28] treten Wundheilungsstörungen und Infektionen seltener auf, da die Gefäße der ventralen Bauchwandhaut geschont werden. Eine Möglichkeit, die weite subkutane epifasziale Mobilisierung zu umgehen, stellt die posteriore Komponentenseparation [2] bzw. der Musculus-transversus-abdominis-Release (TAR) dar.

Eucker et al. [7] beschrieben 2017 ein innovatives Vorgehen zur Behandlung großer Bauchwandhernien. Das Vorgehen wurde als „abdominal wall expander system“ (AWEX) bezeichnet und dehnt durch einen nach ventral gerichteten Zug die Bauchwand derart, dass ein direkter Faszienverschluss ermöglicht wird. Eickhoff et al. [5] zeigten in einem porcinen In-vivo-Modell, dass durch einen ventral gerichteten Zug die Bauchdecke bei vorhandenem Laparostoma derart gedehnt werden kann, dass es zu einer signifikanten Verringerung der notwendigen Verschlusskraft des Abdomens kommt. Mit diesem Verfahren steht eine potenziell schonendere Alternative zu den bisher eingesetzten Methoden zur Verfügung.

Nach tierexperimentellen Untersuchungen [5], Fallberichten [8–11] und einer kleineren Beobachtungsstudie [23] sollen in dieser Studie die Effektivität, klinische Praktikabilität und Komplikationsrate der intraoperativen Faszientraktion (IFT) bei der Behandlung großer ventraler Hernien an einem größeren Patientenkollektiv untersucht werden.

## Methoden

### Patientenauswahl

Um ein Selektionsbias zu vermeiden, wurde allen Patienten mit komplexen Bauchwandhernien während des Erhebungszeitraums die IFT angeboten. Über dieses Verfahren sowie über Alternativverfahren wie die Komponentenseparation wurde ausführlich aufgeklärt. Alle Patienten, die der IFT zugestimmt haben, wurden mit diesem Verfahren behandelt.

Bei allen Patienten wurde zunächst der Versuch des spannungsarmen Direktverschlusses unternommen. Patienten mit möglichem Direktverschluss ohne spezifische operative Maßnahme wurden nicht einbezogen. Bei Unmöglichkeit des Direktverschlusses wurde die IFT eingesetzt.

In die Auswertung flossen die Daten von 50 konsekutiv mit IFT behandelten Patienten ein. Es wurden Patienten aus 11 spezialisierten Zentren im Zeitraum von November 2019 bis April 2021 analysiert. Die Patientendaten wurden dem Herniamed-Register [12] entnommen und dann anonymisiert ausgewertet. Eine Einwilligung der Patienten zur Datenanalyse lag vor.

### Chirurgisches Verfahren

Alle diagnostischen und therapeutischen Maßnahmen erfolgten im Rahmen des klinischen Behandlungsstandards. Die Datendokumentation und -speicherung wurden nach den Regeln nationaler und internationaler Datenschutzverordnungen durchgeführt. Die eingesetzten Medizinprodukte (fasciotens® Hernia/Abdomen, fasciotens GmbH, Essen, Deutschland) sind für die Indikation zugelassen (Fasciotens Hernia, Z/19/0457E Risikoklasse I).

**Figure 1:**
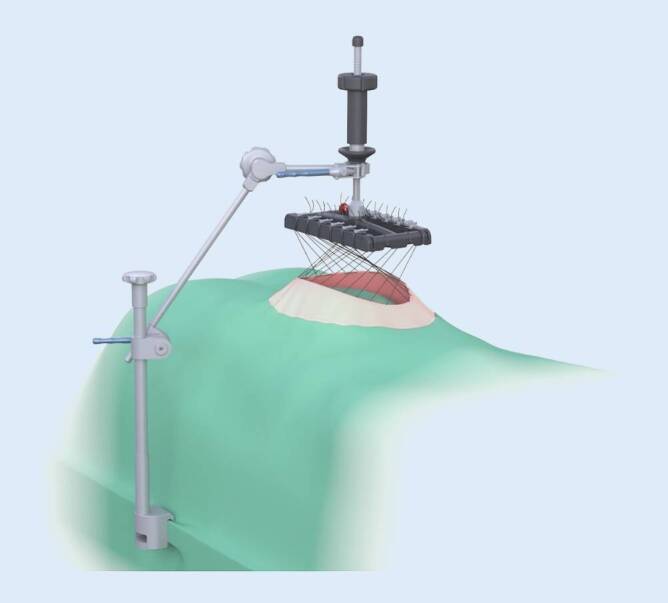

Fasciotens® Hernia/Abdomen dient der Dehnung der Bauchdecke zum primären spannungsarmen Bauchwandverschluss. Hierfür wird über eine externe Vorrichtung ein – nach ventral gerichteter – Zug an die Bauchwandstrukturen angelegt (Abb. 1). Dabei werden in gleichmäßigem Abstand von ca. 2 cm handelsübliche chirurgische Fäden längs in die Faszie mit einem Abstand zum medialen Faszienrand von 1 cm und einer Stichlänge von 2 cm appliziert. Das Nahtmaterial wird dann an einer speziell entwickelten Fadenhalterung des Rahmens befestigt, sodass die Fäden einzeln nachspannbar sind und die kumulativ applizierte Zugkraft ablesbar ist. Mit quantifizierbarer Zugkraft wird die Bauchwand nach ventral gezogen und so ein kontinuierlicher Zug auf die Bauchwand ausgeübt. Der Zug auf die Bauchwand bzw. Faszie kann so lange aufrechterhalten werden, bis ein für den Verschluss der Faszie ausreichender Längengewinn der Bauchwand bzw. eine ausreichende Volumenvergrößerung der Bauchhöhle erreicht ist. Der Zug kann sowohl vertikal als auch diagonal-vertikal ausgerichtet sein. In der vorliegenden Untersuchung wurden alle Patienten für die Dauer von 30–35 min mit einem diagonal-vertikalen Zug von ca. 12 kg behandelt. Während der Traktionsdauer wurden die Fäden alle 2 min nachgespannt.

Es wurde eine Vollrelaxation nach Maßgabe und im Ermessen der Anästhesie durchgeführt. Unmittelbar vor der prätraktionalen Hernienweitenmessung wurde durch die Anästhesie erneut relaxiert.

In 7 Fällen mit teilweise transkutan eingebrachten Zugfäden war die Präparation des retromuskulären Raumes nach den Mini-(to)-less-open-sublay(MILOS)-Prinzipien transhernial mit Eröffnung der hinteren Blätter der Rektusscheiden erfolgt. Der hierzu notwendige Hautschnitt war nicht länger als der Bruchringdurchmesser. Um eine perkutane Zugfädenplatzierung in den vorderen Blättern der Rektusscheiden durchzuführen, war eine sparsame subkutane Präparation notwendig. Dieses Manöver ermöglicht es, selbst bei großen W3-Bruchlücken mit einer relativ kleinen Hautinzision auszukommen. In allen Fällen wurde sowohl beim „weit offenen“ als auch beim teilweise transkutanen Vorgehen ein mindestens 20 × 30 cm messendes Netz in Sublay-Position eingebracht. Insgesamt wurden 42 Patienten mit einem Cicat/Dynamesh (Dahlhausen GmbH Siegburg, Deutschland), 3 Patienten mit Softmesh/Ethicon (Ethicon J&J Medical Devices, Norderstedt, Deutschland), 3 Patienten mit Softmesh/BD (BD GmbH, Heidelberg, Deutschland) und 2 Patienten mit Optilene elastic/BBraun (B.Braun SE, Melsungen, Deutschland) jeweils in Sublay-Position augmentiert.

Von den 45 Patienten, bei denen nach IFT ein Direktverschluss möglich war, wurden 41 Patienten mit einem in retromuskulärer Sublay-Position in der Rektusloge eingebrachten Netz versorgt. Bei 4 weiteren Patienten mit möglichem Direktverschluss erfolgte zusätzlich ein Musculus-transversus-abdominis-Release (TAR) aufgrund zusätzlicher Pathologien (z. B. zusätzliche Hernie an ehemaliger Stomaausleitung).

Bei den 5 Patienten, bei denen ein Direktverschlusses nach IFT nicht möglich war, wurde zusätzlich ein TAR durchgeführt. Bei 3 Patienten war daraufhin ein direkter Verschluss möglich.

Patienten mit möglichem Direktverschluss nach IFT und TAR (*n* = 7) erhielten eine Netzaugmentation in Sublay-Position sowohl in den Rektuslogen als auch auf der jeweiligen Transveralisfaszie hinter dem Transversalismuskel.

Von den beiden Extremfällen mit Bruchweiten von 35 und 44 cm wird hier einer exemplarisch gesondert beschrieben: Bei der Patientin mit der Bruchweite von 35 cm fand sich ein großer Narbenknochen. Hier wurde nach Resektion des Narbenknochens der verbliebene peritoneale Defekt mit einem offen eingenähten 12 cm messenden IPOM(intraperitoneale Onlay-Mesh-Technik)-Netz verschlossen. Das darüber in Sublay-Position eingebrachte Netz maß 30 × 25 cm. Die verbliebene Lücke zwischen den beiden vorderen Blättern der Rektusscheiden betrug nach IFT 19 cm, nach zusätzlicher TAR 12 cm und wurde durch ein Bridging-Netz von 15 × 22 cm verschlossen (Dreifachsandwich).

Der zweite Fall mit der Bruchweite von 44 cm wurde ähnlich versorgt – mit Ausnahme des bei diesem Patienten nicht notwendigen IPOM-Netzes.

### Statistik

Die Daten wurden als Einzelmesswerte, Mittelwerte ± SEM („standard error of the mean“) sowie Mediane (Range) dargestellt. Es wurde der Wilcoxon-matched-pairs-signed-ranks-Test durchgeführt (GraphPad InStat, San Diego, USA). Ein *p* < 0,05 wurde als statistisch signifikant gewertet.

## Ergebnisse

### Patientencharakteristika

Das Durchschnittsalter der Patienten betrug 60,4 ± 2,1 Jahre. Mit einem durchschnittlichen Body Mass Index von 30,5 ± 0,9 kg/m^2^ waren die meisten Patienten übergewichtig. Die Patienten hatten – bis auf einen Patienten mit ASA (American Society of Anesthesiologists) Score I – einen ASA Score von II–III.

Die Patientencharakteristika sind in Tab. 1 zusammengefasst.

**Table 1:** 

**1. Patientencharakteristika**	
*Geschlecht (männlich/weiblich)*	20/30
*Alter (Jahre)*	
Mittelwert ± SEM	60,4 ± 2,1 (*n* = 49)
Median, Range	59 (33–89)
*Body Mass Index (kg/m*^*2*^*)*	
Mittelwert ± SEM	30,5 ± 0,9
Median, Range	30,4 (20,3–49,1)
*ASA*	
I	1
II	29
III	20
IV	0
**2. Faszienmessungen**	
*Faszienabstand vor IFT (cm)*	
Mittelwert ± SEM	16,1 ± 0,8
Median, Range	15 (8–44)
*Faszienabstand nach IFT (cm)*	
Mittelwert ± SEM	5,8 ± 0,7
Median, Range	3,5 (0–19)
*Reduktion des Faszienabstands (cm)*	
Mittelwert ± SEM	10,2 ± 0,7
Median, Range	9 (0–26)
**3. Chirurgische Ergebnisse**	
*Verschlussrate*	45/50 (90 %)
*Operationsdauer (min)*	
Mittelwert ± SEM	207,3 ± 11,0
Median, Range	182,5 (95–390)
*Postoperative Komplikationen*	6/50 (12 %)
*Reoperationen*	3/50 (6 %)
*Krankenhausaufenthaltsdauer (Tage)*	
Mittelwert ± SEM	8,8 ± 1,4
Median, Range	6 (2–73)

*IFT* intraoperative Faszientraktion, *SEM* „standard error of the mean“, *ASA* „American Society of Anesthesiologists physical status classification system“

Unter den versorgten Hernien fanden sich 48 Narbenhernien und 2 epigastrische Primärhernien. In 46 Fällen war die Hernie nach einer Medianlaparotomie, in 2 Fällen nach einer Querlaparotomie entstanden.

### Faszienmessungen

Die Durchführung der IFT mit dem fasciotens® Hernia/Abdomen-Verfahren wurde in 11 verschiedenen Zentren durchgeführt und erwies sich als praktikabel.

**Figure 2:**
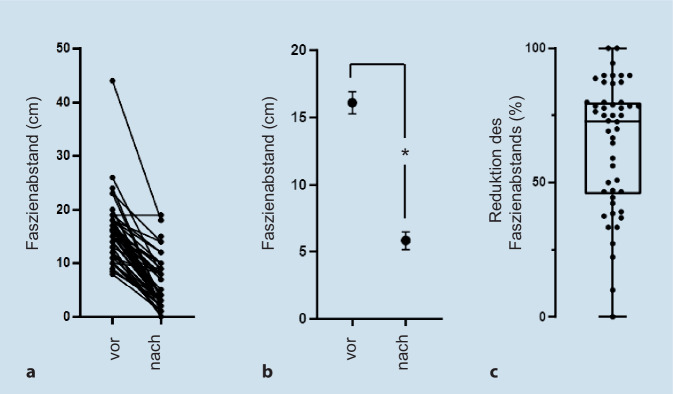

Die präoperativ gemessenen Faszienabstände betrugen 8–44 cm, wobei die meisten Patienten (94 %) einen Faszienabstand größer als 10 cm aufwiesen (W3 nach European Hernia Society). Mit einer Ausnahme konnte bei allen Patienten durch die IFT der Faszienabstand deutlich verringert werden (Abb. 2a). Der durchschnittliche Faszienabstand wurde signifikant von 16,1 ± 0,8 auf 5,8 ± 0,7 cm reduziert (*p* < 0,0001, Abb. 2b). Die erreichte mittlere Reduktion des Faszienabstands war beträchtlich und betrug durchschnittlich 10,2 ± 0,7 cm. Bei drei Viertel der Patienten konnte eine Reduktion des Faszienabstands um mindestens 50 % erreicht werden, und bei der Hälfte der behandelten Patienten betrug die Reduktion des Faszienabstands sogar über 70 % (Abb. 2c und 3). Lediglich bei 3 von 50 Patienten betrug die Abstandsreduktion weniger als 25 %. Die Faszienmessungen sind in Tab. 1 zusammengefasst.

**Figure 3:**
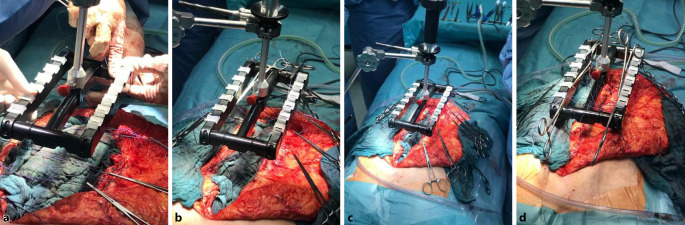

### Chirurgische Ergebnisse

Die durch IFT nach einer mittleren Operationsdauer von 207,3 ± 11,0 min erreichte Verschlussrate war mit 90 % (45/50) hoch. Postoperative Komplikationen traten bei 6 Patienten auf (12 %). Bei 3 Patienten (6 %) war jeweils eine Reoperation erforderlich.

Von den 6 Fällen mit Komplikationen zeigten 2 Patienten ein sonographisch nachgewiesenes subkutanes Serom, das nicht punktiert werden musste und nach jeweils 3 Monaten in einer erneuten Ultraschalluntersuchung deutlich rückläufig war. In 1 Fall sahen wir ein organisiertes subkutanes Hämatom, das ebenfalls konservativ behandelt wurde und nach 3 Monaten sonographisch deutlich kleiner imponierte.

In den 3 behandlungsbedürftigen Fällen musste in 2 Fällen eine Unterdrucktherapie (VAC) aufgrund einer subkutanen Wundheilungsstörung (infiziertes Hämatoserom) eingeleitet werden. In einem Fall waren 11 VAC-Wechsel bis zum sekundären Verschluss von Haut und subkutanem Gewebe notwendig. Im zweiten Fall genügte 1 VAC-Wechsel. In einem Fall wurde eine 2 × 3 cm große Hautnekrose exzidiert. Anschließend erfolgte ein direkter Hautverschluss. Die eigentliche Rekonstruktion der tiefen Bauchwandschichten inklusive der in Sublay-Position eingebrachten Netze war in keinem Fall betroffen und musste in keinem Fall aufgelöst werden.

In 49 Fällen erfolgten die Primärverschlüsse mittels Hautnaht oder Klammern. In 1 Fall wurde postoperativ ein subkutaner, epifaszialer VAC angelegt. Alle Patienten erhielten eine Drainage im Netzlager, bei einigen Patienten wurde eine zusätzliche subkutane Drainage angelegt.

Die durchschnittliche Krankenhausaufenthaltsdauer war mit 8,8 ± 1,4 Tagen (2–73) niedrig. Die Ergebnisse sind in Tab. 1 zusammengefasst.

## Diskussion

Die vorliegende Untersuchung bestätigt und erweitert die bisherigen Einzelfallberichte [8–10, 23] und zeigt eine hohe Effektivität der IFT bei guter klinischer Praktikabilität und niedriger Komplikationsrate.

Die Messbarkeit der Zugkräfte stellt eine entscheidende Weiterentwicklung der von Eucker et al. [7] eingeführten Traktionsmethode dar und ermöglicht die Applikation einer standardisierten Zugkraft an den Faszien. So wird eine ausreichende Zugkraft gewährleistet und ein übermäßiger Zug verhindert. Mit einer erreichten Verschlussrate von 90 % bei großen (mittlerer Herniendurchmesser 16,1 cm) Hernien war die Effektivität der intraoperativen Faszientraktion hoch. Die Methode erwies sich als praktikabel, die durchschnittliche Operationsdauer lag unter 4 h und war somit mit den Komponentenseparationsverfahren vergleichbar. Die 30- bis 35-minütige IFT hat somit nicht zu einer Verlängerung der Operationszeit geführt.

Durch die IFT kam es zu einem individuell unterschiedlichen Ausmaß an Reduktion der Faszienabstände. Wenngleich eine statistische Subgruppenanalyse aufgrund der relativ niedrigen Fallzahl nicht durchgeführt werden konnte, entstand der klinische Eindruck, dass die besten Effekte bezüglich der Abstandsreduktion der Faszien bei Frauen im Unter- und Mittelbauch bestanden. Es ist zu vermuten, dass bei muskelstarken Männern die Reduktion des Faszienabstands durch die IFT niedriger sein könnte als bei Frauen. Die geringeren Effekte im Epigastrium könnten auf die kürzere zu dehnende Bauchwand im Bereich der Rippen zurückgeführt werden.

Bei den verschiedenen Verfahren der Komponentenseparation [6, 16–18, 24] wird die Integrität der lateralen Bauchwand nicht gewahrt, was zu Durchblutungsstörungen durch Durchtrennung der Perforatorgefäße führen kann [24]. Aufgrund der Invasivität dieser Verfahren sind Komplikationen (Serome, Infekte, Hämatome, Bauchwandnekrosen, Sensibilitätsstörungen) nicht selten [16, 17].

**Figure 4:**
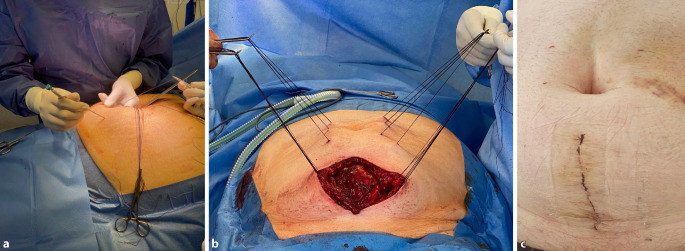

Durch eine reine Dehnung der Bauchdecke bei IFT bleibt die laterale Bauchwand intakt, und es kommt zu keiner lokalisierbaren Schwächung der Faszien. Mechanistisch ist von der zumindest teilweisen Rekonstitution der kontrahierten lateralen Bauchwandmuskulatur auszugehen, die im Rahmen des chronischen Kontinuitätsverlustes der abdominellen Muskelschlinge entstanden ist. Für die Platzierung der Zugfäden ist nur eine sparsame Präparation der Faszie und des Subkutangewebes notwendig, sodass die subkutane Wundfläche zumindest im Vergleich zur Technik nach Ramirez deutlich kleiner ist. Eine teils offene, teils transkutane Fadenplatzierung ist möglich, sodass auch die intraoperative Faszientraktion mit den Prinzipien der MILOS-Technik kombiniert werden kann (Abb. 4).

Maloney et al. [19] berichteten über eine Komplikationsrate von 43 % nach anteriorer bzw. von 31 % nach posteriorer Komponentenseparation. In einer Metaanalyse, basierend auf 63 Studien, errechneten Switzer et al. [30] eine totale Wundinfektionsrate von 21 % für minimal-invasive oder endoskopische Komponentenseparationen und von 35 % für offene Komponentenseparationen. Ähnliche Ergebnisse wurden auch in der auf 7 Studien basierenden Metaanalyse von Hodgkinson et al. [13] publiziert. Eine retrospektive Kohortenstudie von Parent et al. [26] zeigte eine Reoperationsrate von 19 % nach minimal-invasiver anteriorer Komponentenseparation und von 12 % nach TAR.

Bei den hier vorgestellten Fällen kam es nach der IFT zu keinen intraabdominellen Komplikationen. Die Gesamtkomplikationsrate von 12 % und davon eine Reoperationsrate von 6 % erscheinen im Vergleich zu den Techniken der Komponentenseparation niedrig und können am ehesten auf die geringere Invasivität der IFT zurückgeführt werden. Die in unserer Studie beobachtete Gesamtkomplikationsrate der IFT ist hierbei vergleichbar mit der Komplikationsrate bei retromuskulären Netzverfahren mit Fasziendefekten größer 100 cm^2^, die 16 % betrug [20].

Die Rekonstruktion komplexer Hernien erfordert für einen anatomischen Verschluss der Bauchwand einen Streckengewinn der Faszie. Ohne einen ausreichenden Streckengewinn kommt es nach erzwungenem Bauchdeckenverschluss häufig zur Erhöhung des intraabdominellen Druckes mit der möglichen Folge eines abdominellen Kompartmentsyndroms, falls der Bauchdeckenverschluss ohne Zusatzverfahren überhaupt gelingt. Wie in der Voruntersuchung [23] kam es in der vorliegenden Studie nach IFT in keinem Fall zu einem postoperativen abdominellen Kompartmentsyndrom.

Die Daten zeigen eine hohe Effektivität der IFT bei guter klinischer Praktikabilität und niedriger Komplikationsrate.

### Limitationen

Folgende Limitationen sollten bedacht werden:

- Es handelt sich um eine nicht kontrollierte, nicht randomisierte und nicht verblindete retrospektive Untersuchung an einem nicht selektierten Kollektiv. Die daraus resultierenden methodischen Probleme limitieren die Aussagekraft. Aufgrund der Beteiligung von mehreren Zentren bestehen hausspezifische Charakteristika in der prä- und postoperativen Versorgung sowie bei den Operationstechniken.
- Über mögliche Langzeitkomplikationen und Rezidivhäufigkeit liegen noch keine Ergebnisse vor.
- Eine vergleichende Untersuchung hinsichtlich Effektivität und Komplikationsrate der IFT im Vergleich zur Komponentenseparation wird die genauere Bewertung des Verfahrens ermöglichen.

## Fazit für die Praxis

- Mit der beschriebenen intraoperativen Faszientraktion (IFT) steht ein neues Verfahren für die Bauchwanddehnung beim offenen Abdomen und bei großen Narbenhernien zur Verfügung.
- Das Medizinprodukt (fasciotens® Hernia) ist für diese Indikation zugelassen (kein Off-label-Gebrauch).
- Die vorliegende Untersuchung zeigt eine hohe Effektivität der IFT bei guter klinischer Praktikabilität und niedriger Komplikationsrate.
