# Erratum zu: Intraoperative Faszientraktion (IFT) zur Behandlung großer ventraler Hernien

**DOI:** 10.1007/s00104-022-01607-w

**Published:** 2022-02-22

**Authors:** Henning Niebuhr, Zaid Omar Malaibari, Ferdinand Köckerling, Wolfgang Reinpold, Halil Dag, Dietmar Eucker, Thomas Aufenberg, Panagiotis Fikatas, René H. Fortelny, Jan Kukleta, Hansjörg Meier, Christian Flamm, Guido Baschleben, Marius Helmedag

**Affiliations:** 1 Hamburger Hernien Centrum, Eppendorfer Baum 8, 20249 Hamburg, Deutschland; 2 Faculty of Medicine, Department of Surgery, University of Tabuk, Tabuk, Saudi-Arabien; 3 Zentrum für Hernienchirurgie, Vivantes Humboldt Klinikum, Berlin, Deutschland; 4 Chirurgische Klinik Kantonsspital Baselland Bruderholz, Bruderholz, Schweiz; 5 Klinik für Chirurgie, St. Elisabeth-Krankenhaus Köln, Köln, Deutschland; 6 Klinik für Chirurgie, Charité Campus Virchow-Klinik, Berlin, Deutschland; 7 Ordinationszentrum der Confraternität, Wien, Österreich; 8 Klinik für Chirurgie, Hirslanden Klinik, Zürich, Schweiz; 9 Klinik für Allgemein- und Viszeralchirurgie, Sana Krankenhaus, Benrath, Deutschland; 10 Klinik für Allgemein‑, Viszeral‑, Endokrine und Unfallchirurgie, RoMed Clinic, Bad Aibling, Deutschland; 11 Klinik für Allgemein- und Viszeral Chirurgie, St. Elisabeth Hospital, Leipzig, Deutschland; 12 Klinik für Allgemein‑, Viszeral- und Transplantationschirurgie, Universitätsklinik Aachen, Aachen, Deutschland

**Erratum zu:**

**Chirurg 2022**

10.1007/s00104-021-01552-0

Der Artikel *Intraoperative Faszientraktion (IFT) zur Behandlung großer ventraler Hernien* von Henning Niebuhr et al. wurde ursprünglich am 12.14.2021 ohne „Open Access“ online auf der Internetplattform des Verlags publiziert. Die Autoren haben sich jedoch nachträglich für eine „Open Access“-Veröffentlichung entschieden. Das Urheberrecht des Artikels wurde in © Der/die Autor(en) 2022 geändert. Der Artikel wird nun unter der Creative Commons Namensnennung 4.0 International-Lizenz veröffentlicht, welche die Nutzung, Vervielfältigung, Bearbeitung, Verbreitung und Wiedergabe in jeglichem Medium und Format erlaubt, sofern Sie den/die ursprünglichen Autor(en) und die Quelle ordnungsgemäß nennen, einen Link zur Creative-Commons-Lizenz beifügen und angeben, ob Änderungen vorgenommen wurden.

Die in diesem Artikel enthaltenen Bilder und sonstiges Drittmaterial unterliegen ebenfalls der genannten Creative-Commons-Lizenz, sofern sich aus der Abbildungslegende nichts anderes ergibt. Sofern das betreffende Material nicht unter der genannten Creative-Commons-Lizenz steht und die betreffende Handlung nicht nach gesetzlichen Vorschriften erlaubt ist, ist für die oben aufgeführten Weiterverwendungen des Materials die Einwilligung des jeweiligen Rechteinhabers einzuholen.

Weitere Details zur Lizenz entnehmen Sie bitte der Lizenzinformation auf http://creativecommons.org/licenses/by/4.0/deed.de.

